# A Supramolecular Hydrogel Enabled by the Synergy of Hydrophobic Interaction and Quadruple Hydrogen Bonding

**DOI:** 10.3390/gels8040244

**Published:** 2022-04-14

**Authors:** Liangmei Lu, Wen Zhou, Zhuzuan Chen, Yang Hu, Yu Yang, Guangzhao Zhang, Zhuohong Yang

**Affiliations:** 1 College of Materials and Energy, Guangdong Laboratory for Lingnan Modern Agriculture, South China Agricultural University, Guangzhou 510642, China; liangmei1010@stu.scau.edu.cn (L.L.); 20203138220@stu.scau.edu.cn (Z.C.); huyang0303@scau.edu.cn (Y.H.); 2 Department of Neurosurgery, The Second Affiliated Hospital, Medical College of Shantou University, 69 North Dongxia Road, Shantou 515041, China; wenzhou@stu.edu.cn; 3 Department of Materials Science & Engineering, Guangdong Provincial Key Laboratory of Energy Materials for Electric Power, Southern University of Science and Technology, Shenzhen 518055, China

**Keywords:** supramolecular self-assembly, self-healing, injectable, synergetic effect, quadruple hydrogen bonding

## Abstract

The increasing preference for minimally invasive surgery requires novel soft materials that are injectable, with rapid self-healing abilities, and biocompatible. Here, by utilizing the synergetic effect of hydrophobic interaction and quadruple hydrogen bonding, an injectable supramolecular hydrogel with excellent self-healing ability was synthesized. A unique ABA triblock copolymer was designed containing a central poly(ethylene oxide) block and terminal poly(methylmethacrylate) (PMMA) block, with ureido pyrimidinone (UPy) moieties randomly incorporated (termed MA-UPy-PEO-UPy-MA). The PMMA block could offer a hydrophobic microenvironment for UPy moieties in water and thus boost the corresponding quadruple hydrogen bonding interaction of Upy–Upy dimers. Owing to the synergetic effect of hydrophobicity and quadruple hydrogen bonding interaction, the obtained MA-UPy-PEO-UPy-MA hydrogel exhibited excellent self-healing properties, and injectable capability, as well as superior mechanical strength, and therefore, it holds great promise in tissue engineering applications, including in cell support and drug release.

## 1. Introduction

Supramolecular hydrogels with excellent self-healing and injectable properties hold great promise in biomedical engineering applications, including in minimally invasive surgery, drug release, and tissue repair [1,2,3,4,5]. This is due to the crosslinking strategies of supramolecular hydrogels, giving them multiple desirable properties such as self-healing, mechanical strength, and simulative responsibility [6,7,8,9]. Developing new supramolecular hydrogels with multiple crosslinking interactions will endow them with many intriguing properties. Physical and chemical crosslinking are the two main strategies to construct the three-dimensional hydrogel networks from linear supramolecular polymers in water environments [10,11,12,13]. Chemical crosslinking has been widely used to prepare tough hydrogels with outstanding mechanical properties due to their relatively high strength [14,15,16,17]. However, most chemical bonds are irreversible, which means that the recovery of the crosslinking between two polymer chains is impossible once it is broken under external force, thus limiting their application as self-healing materials [18,19,20]. Unlike chemical bonds, the crosslinking between hydrophilic polymers via physical interactions, such as π–π stacking, hydrogen bonding, electrostatic force, host–guest interaction, and hydrophobicity, can reform and regain their physical or chemical characteristics after being destroyed by an external force due to their self-healing capability [21,22,23,24,25]. Incorporating two or more non-covalent crosslinking interactions into water-soluble polymers with well-designed synergetic effects will endow these newly prepared hydrogels with multiple tunable properties and thus broaden their wide application in many fields [26,27,28,29]. For instance, Christopher et al. introduced cyclodextrin, adamantane, and acrylates into gelatin chains and prepared dual network hydrogels with excellent self-healing and injectable properties for tissue repair.

Ureido pyrimidinone (UPy) moieties, which were first investigated by Meijer et al., can formulate self-complementary dimers through quadruple hydrogen bonding interactions, meaning they can be widely used to assemble supramolecular polymers with self-healing capability and, recently, multifunctional hydrogels [30,31,32]. More importantly, Meijer et al. have found that the strong interaction of UPy hydrogen bonds can only maintain in hydrophobic microenvironments, and the dimerization constant decreases dramatically when the environment becomes hydrophilic, which is probably ascribed to the competition of water molecules, which can form stable hydrogen bonds with UPy moieties [33]. Given that the hydrogel supramolecular polymer chains are surrounded by water, the introduction of hydrophobic segments around the UPy moieties is necessary to prepare UPy-based hydrogels with robust mechanical properties [34,35].

In this study, we demonstrate a well-designed supramolecular hydrogel with a synergetic effect of hydrophobic interactions and UPy dimerization. The as-assembled supramolecular hydrogel is flexible and tunable. This is due to the utilization of a water-soluble ABA tri-block copolymer containing a central poly(ethylene oxide) block (A) and terminal poly(methylmethacrylate) (PMMA) block with ureido pyrimidinone (UPy) moieties randomly incorporated (B), which was synthesized by reversible additional fragment transfer (RAFT) polymerization (termed MA-UPy-PEO-UPy-MA). The PMMA block plays two important roles here: (1) it offers a hydrophobic microenvironment for UPy moieties and subsequently enhances the strength of quadruple hydrogen bonds, and (2) it facilitates the supramolecular self-assembly of the hydrogel via hydrophobic interactions [36,37]. Due to the unique synergetic effect of PMMA and UPy, the assembled hydrogels reveal a quick self-healing capacity after mechanical damage, and they can be injected through syringes with small gauges. Moreover, drug release tests using the supramolecular as carriers suggest the potential application of the prepared supramolecular hydrogels in biomedical engineering. These unique properties will be significantly beneficial to minimally invasive surgery, as the hydrogels are convenient to handle and can be transplanted by injection.

## 2. Results and Discussion

### Design of Supramolecular Hydrogel via Synergetic Effect of Hydrophobicity and Quadruple Hydrogen Bonding

**Figure 1:**
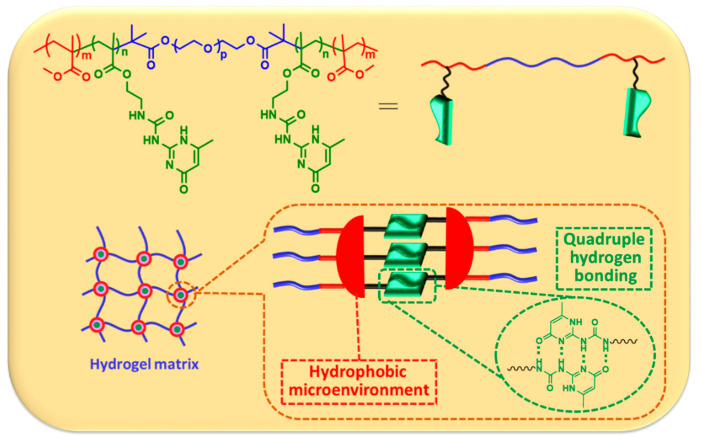
The design of supramolecular hydrogel based on the synergy between hydrophobic interaction and quadruple hydrogen bonding.

Due to the competition effect of water molecules, the quadruple hydrogen bonding strength of UPy dimers is considerably weakened in hydrophilic environments [38,39]. Given that hydrogel is composed of hydrophilic polymer chains with a large amount of encapsulated water, a hydrophobic microenvironment that can protect the UPy moieties from direct exposure to water is necessary to enhance the dimerization constants [40,41]. Therefore, we designed an ABA triblock copolymer with a central poly(ethylene oxide) block (PEO, A, hydrophilic part) and terminal poly(methylmethacrylate) (PMMA) block with UPy moieties randomly incorporated (B), which was synthesized by reversible additional fragment transfer (RAFT) polymerization (termed MA-UPy-PEO-UPy-MA), as demonstrated in Figure 1. The center PEO part is necessary to keep the hydrophilic property of the whole supramolecular polymer, which is indispensable for hydrogel preparation. It is worth noting that the terminal PMMA segment was designed as an essential part to provide a hydrophobic microenvironment for UPy moieties, which subsequently enhances the quadruple hydrogen bonding strength and facilitates the supramolecular self-assembly of the hydrogel with the protection of PMMA block, and the UPy–UPy dimer interaction is effectively enhanced, which determines the final crosslinking of the supramolecular hydrogels.

**Figure 2:**
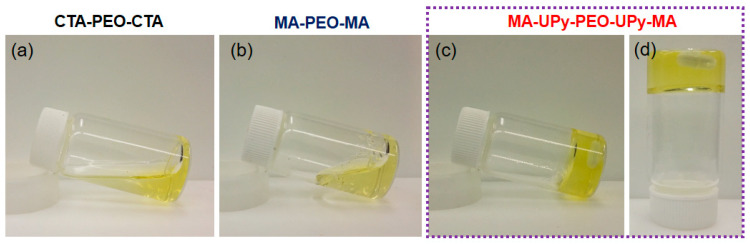
The gestation test of supramolecular hydrogel: (**a**,**b**) the solution of CTA-PEO-CTA and MA-PEO-MA is at a sol state at room temperature, while the solution of MA-UPy-PEO-UPy-MA can form a stable hydrogel (**c**,**d**).

The unique ABA triblock copolymer was synthesized via RAFT polymerization (MAUPy-PEO-UPy-MA), while corresponding copolymers without UPy and/or PMMA segments were also synthesized for comparison (CTA-PEO-CTA and MA-PEG -MA, Scheme S1, Figures S1–S4). The chemical structures of the prepared supramolecular copolymers were first identified by proton nuclear magnetic resonance (NMR). As shown in Figure S5, the characteristic peaks around 2.9 ppm in the spectra of MA-PEG-MA and MA-UPy-PEO-UPy-MA were attributed to methyl groups in the PMMA segment, suggesting the copolymerization of MA into the CTA-PEO-CTA macromolecule chain transfer reagent. Compared with MA-PEG-MA, three small peaks around 10.6, 12.1, and 13.2 ppm in the spectrum of MA-UPy-PEO-UPy-MA were clearly observed, which strongly suggests the successful incorporation of UPy moieties in the supramolecular polymer chains. Noting that the peak around 0.85 ppm was associated with the methyl groups in the CTA-PEO-CTA chain transfer reagent, we then calculated the repeated unit number of MA and UPy moieties to be 15 and 2 according to the area integral of corresponding peaks, respectively. The gelation performance of the supramolecular hydrogel is of great significance to its further application in biomedical engineering [42,43]. We then characterized the gelation behavior using the simple tilting test with the polymer concentration of 10 wt%, while the corresponding polymer solutions were prepared by dissolving the copolymers into deionized water under stirring at 60 ℃. As shown in Figure 2a, the solution of CTA-PEO-CTA exhibited a liquid state at room temperature, suggesting that almost no crosslinking interactions existed between polymer chains. After the copolymerization of MA, the obtained MA-PEG-MA solution was a liquid with high viscosity but still could flow after bottle tilting (Figure 2b). This hints that the hydrophobic interaction of the PMMA segment can offer physical crosslinking to the polymers, but this interaction is too weak to trigger the construction of a three-dimensional polymer network. Interestingly, after incorporating the UPy moieties, the MA-UPy-PEO-UPy-MA solution showed a stable gel state at room temperature, and this gel could even maintain its form after reversing the glass bottle (Figure 2c). This phenomenon suggests that the strong interaction of UPy quadruple hydrogen bonding is strong enough to crosslink the polymer chains, thus triggering the gelation of the polymer solution. From the gelation test, we concluded that the strong quadruple hydrogen bonding of UPy moieties is the key reason why supramolecular hydrogels crosslink polymers to form 3D networks, while the PMMA segment offers a hydrophobic microenvironment to UPy moieties. The synergetic effect of hydrophobic interaction and the quadruple hydrogen bonding facilitates the formation of supramolecular hydrogels.

**Figure 3:**
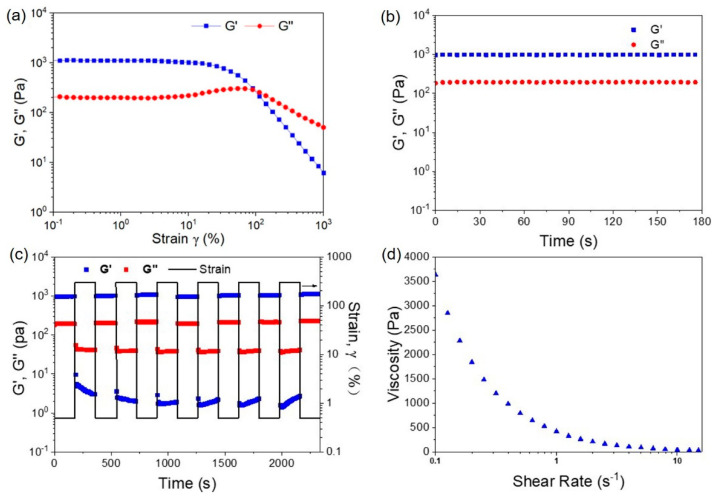
Rheological characterization of obtained hydrogel: (**a**) strain-sweep measurements; (**b**) an immediate recovery after the 1000% strain deformation; (**c**) dynamic strain amplitude cyclic test (γ = 0.5% or 300%); (**d**) viscosity measurement.

The mechanical property of the prepared supramolecular hydrogel was investigated by using rheological measurement, and the results are displayed in Figure 3. Figure 3a depicts the strain–sweep measurements of a 10 wt% MA-UPy-PEO-UPy-MA hydrogel at 25 °C and immediate recovery after the large strain deformation (Figure 3b). It is apparent that both the storage modulus (G′) and the loss modulus (G″) of the MA-UPy-PEO-UPy-MA hydrogel maintained constant values with the increase in strain from 0.1% to 100%, and the value of G′ was much larger than that of G″, confirming the synthesized hydrogel could withstand relatively large deformations. With the further increase in the strain over 200%, both the G′ and G″ decreased dramatically, with the G″ exceeding G′ in value at the strain of 250%, indicating the hydrogel network was destroyed completely by the external force. However, when a relatively small strain (γ = 0.5%) was applied to the MA-UPy-PEO-UPy-MA hydrogel immediately after the removal of large strain deformation, both the G′ and G″ recovered to their initial value without any loss in a few seconds (Figure 3b), demonstrating a remarkable self-healing capacity of the fabricated hydrogel.

Meanwhile, the repeated dynamic strain amplitude cyclic test (γ = 0.5% or 300%) of the MA-UPy-PEO-UPy-MA hydrogel was performed to investigate its self-healing capability. As shown in Figure 3c, when a 300% strain was applied, the G′ of the hydrogel immediately dropped from about 1000 Pa to 40 Pa and became smaller than that of G″ (400 Pa), revealing that the supramolecular hydrogel was disrupted and subsequently turned into a sol state. Interestingly, when a small strain (0.5%) was applied after the removal of the large one, both G′ and G″ immediately recovered to their initial states without any loss, indicating that the hydrogel repaired itself and rebuilt its 3D networks after removing the external force. After repeated testing for five cycles, the hydrogel could still maintain its initial mechanical properties, exhibiting outstanding recovery capacity. This self-healing capability is attributed to the inherent reversibility of quadruple hydrogen bonding interaction from UPy dimers and the unique protection of the PMMA hydrophobic microenvironment. Viscosity measurement on the MA-UPy-PEO-UPy-MA hydrogel was carried out, and an apparent shear-thinning behavior was observed, as shown in Figure 3d. This superior property of the prepared supramolecular hydrogel makes it possible to be injected with a small gauged syringe.

**Figure 4:**
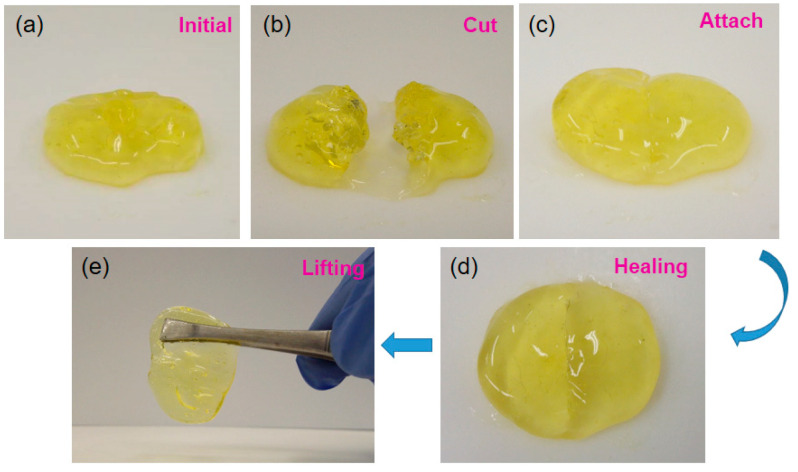
(**a**–**e**) The optical self-healing process of MA-UPy-PEO-UPy-MA supramolecular hydrogel.

The self-healing ability of the prepared MA-UPy-PEO-UPy-MA hydrogel was intuitively characterized using the optical broken-repairing test: One piece of hydrogel was cut with a medical blade and then attached together to check the self-healing behavior. As clearly shown in Figure 4a–e, the damaged hydrogel pieces after cutting could adhere to each other instantly once contacting and simultaneously heal into one integral hydrogel. Moreover, the healed hydrogel was sufficiently strong to withstand its own weight after lifting with a stainless-steel tweezer, suggesting the remarkable self-healing capacity of the MA-UPy-PEOUPy-MA hydrogel. As a soft material with excellent self-healing ability, the self-repairing process after external stimuli is of great significance [44,45].

**Figure 5:**
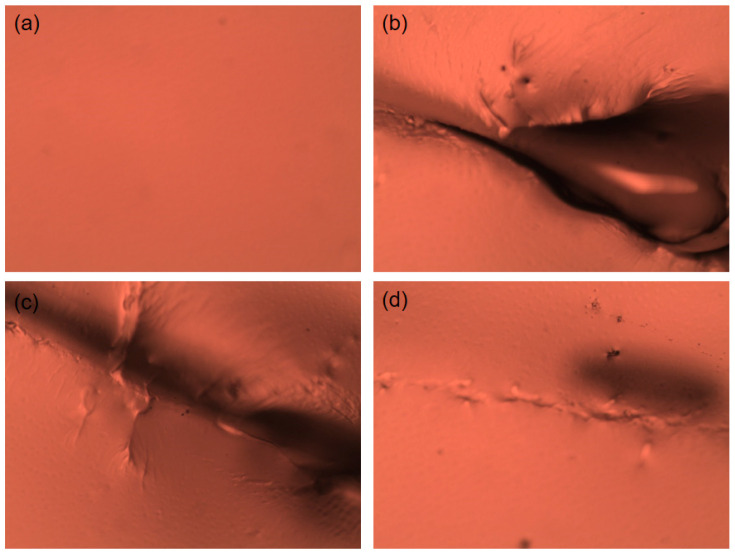
Optical microscope images of the self-healing process of supramolecular hydrogel: (**a**) initial hydrogel; (**b**) damaged hydrogel; (**c**) after healing 0.5 h; (**d**) after 5 h healing.

We then tracked the self-repairing process of the MA-UPy-PEO-UPy-MA hydrogel with an optical microscope. As depicted in Figure 5a, the surface of the supramolecular hydrogel exhibited a flat morphology, and an apparent depredation ditch was observed after cutting with a doctor blade (Figure 5b). After healing for 0.5 h at room temperature, both the depth and the width of the ditch became smaller (Figure 5c), suggesting the gradual repairing of the damaged hydrogel. After healing for another 1 h, the hydrogel healed completely with a flat micromorphology, similar to its initial state (Figure 5d).

**Figure 6:**
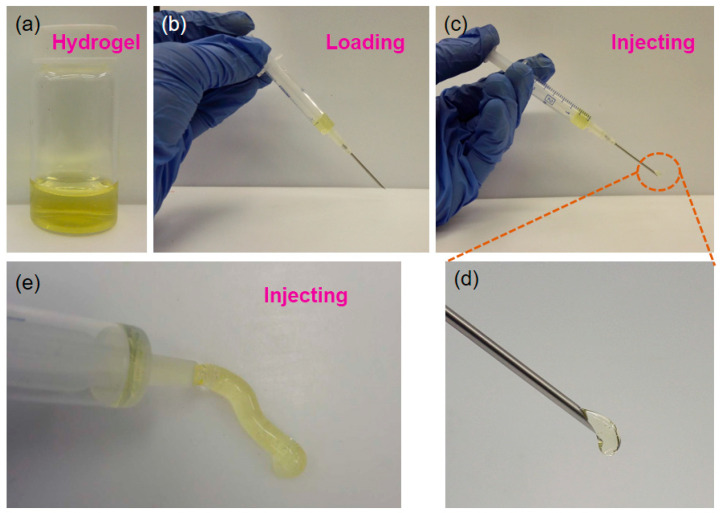
(**a**–**e**) The injectable characterization of supramolecular hydrogel.

As supramolecular hydrogels are self-healing soft materials that hold great promise in biomedical engineering applications, especially for minimally invasive surgery, their injecting ability is vital and significant [46,47,48]. We then characterized the injecting behavior of the prepared MA-UPy-PEO-UPy-MA hydrogel with a syringe equipped with a small-gauged needle (24 gauge). As clearly depicted in Figure 6a, the prepared supramolecular polymer solution was in a gelation state at room temperature and could be facially loaded into the plastic syringe at 60 ℃ because high temperature can destroy hydrogen bonding and the hydrophobic interactions, causing the hydrogel to transform to a sol state (Figure 6b). When applying a relatively small force, the hydrogel could be injected out from a small-gauged needle (Figure 6c,d), suggesting the excellent injecting capability of the MA-UPy-PEO-UPy-MA hydrogel. After injecting from the syringe, the hydrogel kept the cylindrical shape without any change (Figure 6e), indicating the superior mechanical performance of the supramolecular hydrogel.

**Figure 7:**
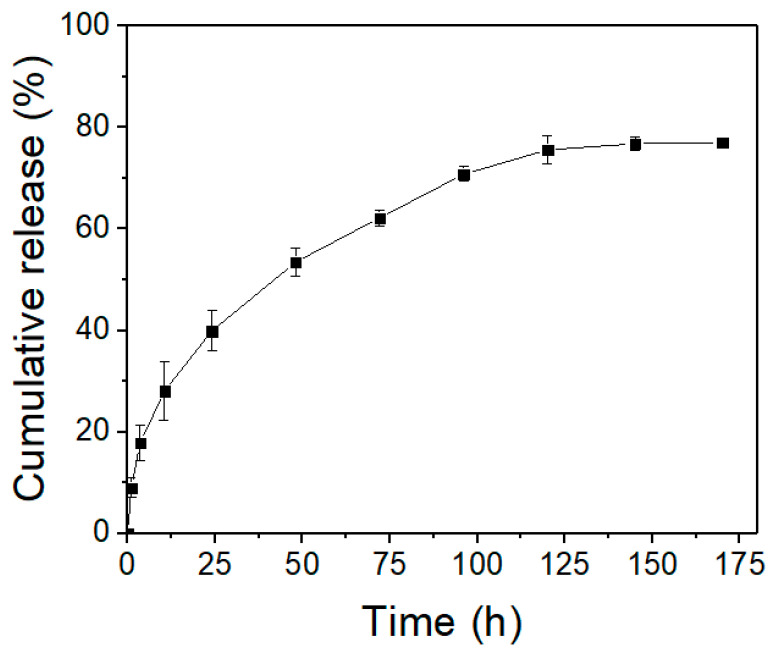
The injectable characterization of supramolecular hydrogel.

It is well known that hydrogels are one of the most promising soft materials used as drug carriers in biomedical engineering applications [49,50,51]. Therefore, the bovine serum albumin (BSA), an important water-soluble protein usually used in biochemical research, was utilized as a target drug for the in vitro drug release test of the as-prepared MAUPy-PEO-UPy-MA supramolecular hydrogel, while the whole release process was detected using UV–Visible absorption spectra. As is shown in Figure 7, the release rate of BSA was relatively fast during the initial 100 h (corresponds to 4 days), and the release amount reached a value of about 75 wt% after another 4 days. The relatively fast release during the initial four days is classic behavior of Fick diffusion, which is probably ascribed to the BSA concentration difference between water and hydrogel network. After the initial fast diffusion, the release rate of the BSA dramatically decreased and stepped into a sustained release state. After the initial 125 h, the in vitro release reached a maximum value of 87 wt%, and the curve shows almost no apparent fluctuation anymore, suggesting that almost all protein was released from the hydrogel and about 13 wt% of target drugs were left in the hydrogel network. Along with its outstanding self-healing capacity and possible potential for injection manipulation, the prepared UPy-PEO-UPy-MA supramolecular hydrogel provided an intriguing glimpse into the application of drug release.

## 3. Conclusions

In summary, we designed and prepared a self-healing supramolecular hydrogel with excellent injectable properties with a synergetic effect of quadruple hydrogen bonding and hydrophobic interaction. The supramolecular MA-UPy-PEO-UPy-MA copolymer was synthesized via RAFT polymerization and identified using 1H NMR. The supramolecular hydrogel was prepared by facile dissolving the prepared polymer into deionized water at 60 °C. The as-prepared hydrogel possessed excellent self-healing property, although with apparent shear-thinning behavior from the rheological measurements. Further rheological investigation of the MAUPy-PEO-UPy-MA hydrogel proved that the quadruple hydrogen bonding interaction of UPy moieties is key to gel formation, while the PMMA segment provides an indispensable hydrophobic microenvironment for UPy. This unique synergetic effect of hydrophobic interaction and quadruple hydrogen bonding interaction endows the prepared hydrogel with outstanding self-healing capability and superior injectable property. Furthermore, the in vitro drug release assay exploiting BSA as a target drug molecule revealed the supramolecular hydrogel to contribute to the sustained release of the good drug. Considering both its outstanding self-healing capacity and tremendous injectability potential, the MA-UPy-PEO-UPy-MA supramolecular hydrogel will be one of the most intriguing candidates in the application of biomedical engineering applications such as tissue repair and drug release.

## 4. Materials and Methods

### 4.1. Materials

For this study, 2-amino-4-hydroxy-6-methyl-pyrimidine was purchased from Sigma-Aldrich and used as received. PEO_445_ (20 KDa), oxalyl chloride ((COCl)_2_), methyl tricaprylylmethylammonium chloride (Aliquot 336), calcium hydride (CaH_2_), and sodium hydroxide (NaOH) were purchased from Aladdin Bio-Chem Technology Co., LTD (Shanghai, China). In addition, 2-isocyanatoethyl methacrylate (MA) and dibutyltin dilaurate (DBTDL) were bought from J&K Scientific Co., Ltd. (Shanghai, China), and 1-Dodecanethiol (CH_3_(CH_2_)_11_SH), carbon disulfide (CS_2_), and calcium chloride (CaCl_2_) were obtained from Fuchen Chemical Reagent Factory (Tianjin, China). Bovine serum albumin (BSA) and xylene brilliant cyanin G were offered by Boao Biotechnology Co. Ltd. (Shanghai, China). All of the other reagents are of analytical grade and used as received, without further retreatment unless specified otherwise. Deionized water was used in all experiments, and 2(6-isocyanatohexylaminocarbonylamino)-6-methyl-4[1H] pyrimidinone (UPy-NCO) was synthesized according to a public method.

### 4.2. Synthesis of UPy-MA Monomer

First, 2-amino-4-hydroxy-6-methyl-pyrimidine (6 g) was completely dissolved in DMSO with heating at 170 °C. The flask was then removed from the oil bath, and 2-isocyanatoethyl methacrylate (8.00 g, 51.6 mmol) was added immediately to the flask. The addition of 2-isocyanatoethyl methacrylate resulted in a vigorous reaction, which was quenched quickly using a water bath to inhibit polymerization. The precipitated white solid that resulted was washed with cyclohexane and dried under reduced pressure to give compound 1 (yield 89%). ^1^H NMR (CDCl_3_, 400 MHz, 298 K) δ 12.95 (s, 1H), 11.96 (s, 1H), 10.51 (s, 1H), 6.16 (s, 1H), 5.77 (s, 1H), 5.55 (s, 1H), 4.26 (t, J = 5.7 Hz, 2H), 3.58 (q, J = 5.7 Hz, 2H), 2.24 (s, 3H), 1.94 (s, 3H).

### 4.3. Synthesis of the RAFT Agent CTA

The agent was synthesized according to the reported method [3]. Generally, a three-neck flask was cooled below 10 °C with a cold circulating pump while removing the containing air with nitrogen. Then, 1-dodecanethiol (12.114 g, 59.85 mmol), acetone (28.86 g, 496.90 mmol), and Aliquot 336 (tricaprylylmethylammonium chloride, 0.9735 g, 2.41 mmol) were added, followed by dropwise adding of sodium hydroxide solution (50 wt%, 5.031 g). The reaction was stirred for another 15 min before carbon disulfide (4.563 g, 60 mmol) in acetone (6.054 g, 103.5 mmol) was added; during this time, the color turned red. Ten minutes later, chloroform (10.688 g, 90 mmol) was added in one portion, followed by dropwise addition of 50% sodium hydroxide solution (24 g, 300 mmol) for 30 min. The reaction was stirred overnight, and 90 mL of water was added, followed by 15 mL of concentrated HCl, to acidify the aqueous solution. Nitrogen gas was purged through the reactor with vigorous stirring to assist the evaporation of acetone. The solid was collected through filtering and then dissolved in 150 mL of 2-propanol. The undissolved solid was filtered off, the remaining 2-propanol solution was concentrated and dried, and the resulting solid was recrystallized from n-hexane, to obtain 13.1 g of yellow crystalline solid (yield: 85%). ^1^H NMR (CDCl_3_): 0.88 (t, 3H, CH_3_CH_2_-), 1.37–1.47 (m, 20H, CH_3_(CH_2_)_10_CH_2_-), 1.75 (s, 6H, -SC(CH_3_)_2_COOH), 3.42 (t, 2H, -CH2S(C=S)).

### 4.4. Synthesis of the Macro-RAFT Agent (CTA-PEO-CTA)

First, CTA (2.0 g, 5.4 mmol) and one drop of DMF were added to a 20 mL of dichloromethane (CH_2_Cl_2_) under a nitrogen atmosphere, followed by sequentially cooling the mixture to 0 °C. After the completed dissolving of CTA, excess oxalyl chloride (3.0 g, 24 mol) was drop added to the solution. The ice bath was removed after the addition of oxalyl chloride, and the reaction proceeded for 2 h at ambient temperature. Excess reagents were then removed under vacuum, and the residue was redissolved in dry CH_2_Cl_2_ (70 mL), followed by the addition of PEG (11 g, 0.54 mmol). The reaction was allowed to proceed for 24 h at room temperature, after which the contents were precipitated in diethyl ether and washed with diethyl ether three times before drying in a vacuum oven. Thus, 9.8 g of light yellow powder was obtained, with a yield of 86%. ^1^H NMR (CDCl_3_): = 4.18 (t, 4H, (C=O)OCH_2_CH_2_O), 3.58 (m, -(CH_2_CH_2_O)n-), 3.20 (t, 4H, -CH_2_S(C=S)), 1.63 (s, 12H, -SC(CH_3_)_2_COOH), 1.19 (m, 40H, CH_3_(CH_2_)_10_CH_2_-), 0.81 (t, 6H, CH_3_CH_2_-).

### 4.5. Synthesis of MA-UPy-PEO-UPy-MA Hydrogel Supramolecular Polymer

The hydrogel polymer MA-UPy-PEO-UPy-MA was synthesized by reversible addition–fragmentation chain transfer polymerization. Briefly, UPy-MA (0.10g, 0.236 mmol) was initially dissolved in 4 mL of DMF in a Schlenk tube under ultrasonication, followed by dissolving CTA-PEO-CTA (1.04 g, 0.05 mmol), MA (1.0 g, 8.85 mmol), and AIBN (2 mg) into this mixture. After three cycles of freeze–pump–thaw, the polymerization was allowed to proceed at 70 °C for 24 h. The polymerization was quenched by adding 6 mL of THF into the above reaction mixture, and the resulting solution was precipitated with the excess amount of diethyl ether. The precipitated copolymer was collected by filtration and then redissolved in 60 mL of deionized water, followed by dialysis against ultrapure water for 7 days. A white powder was finally obtained with a yield of 87% (1.86 g) after freeze-drying. ^1^H NMR was carried out to confirm the copolymer, and the result is shown in Figure S5. A triblock copolymer MA-PEO-MA without UPy functionality was also synthesized as a control. The protocol was identical to that of MA-UPy-PEO-UPy-MA synthesis, except that no UPy-MA monomer was added during the polymerization. Moreover, 1H NMR of MA-PEO-MA was also recorded, which is shown in Figure S5.

### 4.6. Hydrogel Fabrication

The hydrogels or polymer solutions were prepared by facilely dissolving the corresponding copolymers into deionized water at 60 °C under stirring until reaching a polymer concentration of 10.0 wt%. Bubbles were removed under vacuum or stored at 60 °C for 4 h.

### 4.7. Rheological test of the MA-UPy-PEO-UPy-MA Hydrogels

The rheological properties of the fabricated supramolecular hydrogels were measured on an ARG-2 rheometer (TA instruments, Unit-ed States). Stainless steel parallel plates with diameters of 40 mm were employed for the test of all hydrogels, and the gap between plates was set at 1 mm. For the convenience of tests, all hydrogel samples were prepared into disc shapes with two glass plates at 37 °C and were allowed to stand for 10 min for complete gelation while fixing the gap at 1 mm. For the strain–sweep test, dynamic strain amplitude cyclic test, and the viscosity measurement, the temperature was fixed at 37 °C with a hydrogel polymer concentration of 10.0 wt%, and silicone oil was used to prevent water evaporation. For oscillatory strain amplitude sweep measurements, the frequency was kept at 1 rad/s while the strain was raised from 0.1% to 500%. For the characterization of self-healing properties, a dynamic step strain amplitude test was performed between 0.5% and 200% for five cycles while keeping the angular frequency at the constant of 1 rad/s. To achieve viscosity variation in self-healing hydrogel under different shear rates, viscosity measurement was conducted by controlling shear rate varying from 0.1 to 100 rad/s.

### 4.8. Self-Healing Test

Two cylindrical hydrogel samples with diameters of 2 cm were initially cut into four pieces. Any two fragments of hydrogels in different colors were put together immediately and were allowed to heal at 37 °C. A stainless steel tweezer was used to pick up the healed hydrogel for examining the healing results.

### 4.9. Injectable Property Test

The hydrogel samples were injected directly through a syringe equipped with a 26 gauge needle at room temperature.

### 4.10. In Vitro Drug Release Test

To characterize the drug release behavior of the supramolecular hydrogel, bovine serum albumin (BSA) served as a target molecule. Generally, BSA was first dissolved into a certain amount of PBS buffer to obtain a solution with a concentration of 10 mg/mL. Then, the BSA solution was subsequently exploited to prepare a 10.0 wt% hydrogel, with a final protein concentration of 8 mg/mL. For release measurements, typically, 0.5 mL of hydrogel was placed into a glass vial containing 4.5 mL PBS buffer. Then, the vial was incubated in a shaking incubator at 37 °C for subsequent release. Afterward, 0.2 mL of release medium was withdrawn, followed by adding an equal volume of fresh PBS buffer for supplementary at predetermined time intervals. The collected aqueous medium was diluted with 0.8 mL PBS and dyed by adding 5 mL xylene brilliant cyanin G solution (0.1 mg/mL), followed by detection with a U-3010 UV–Vis spectrometer at 595 nm. The release curve was achieved according to the calibration curve established from the standard BSA solution.

## Supplementary Materials

The following supporting information can be downloaded at: https://www.mdpi.com/article/10.3390/gels8040244/s1, Scheme S1: Synthesis of UPy-MA monomer; Figure S1: H NMR spectrum of UPy-NMA monomer; Figure S2: Synthesis of RAFT agent CTA; Figure S3: The H NMR spectrum of CTA; Figure S4: The synthesis of macro-RAFT agent (CTA-PEO-CTA); Figure S5: Characterizing the supramolecular polymers using H NMR.
