# Supplementary material for: A Supramolecular Hydrogel Enabled by the Synergy of Hydrophobic Interaction and Quadruple Hydrogen Bonding
Source: Gels. 2022 Apr 14;8(4):244. doi: 10.3390/gels8040244 (PMC9032949; doi:10.3390/gels8040244)
Supplement: Supplementary file 1 [file gels-08-00244-s001.zip › gels-1627192 - Supplementary material - final (affiliation updated).pdf]

gels

Article

A Supramolecular Hydrogel Enabled by the Synergy of Hydrophobic Interaction and Quadruple Hydrogen Bonding

Liangmei Lu [1,†], Wen Zhou [2,†], Zhuzuan Chen [1], Yang Hu [1], Yu Yang [1,*], Guangzhao Zhang [3,*] and Zhuohong Yang [1,*]

1 College of Materials and Energy, Guangdong Laboratory for Lingnan Modern Agriculture, South China Agricultural University, Guangzhou 510642, China liangmei1010@stu.scau.edu.cn (L.L.); 20203138220@stu.scau.edu.cn (Z.C.); huyang0303@scau.edu.cn (Y.H.)
2 Department of Neurosurgery, The Second Affiliated Hospital, Medical College of Shantou University, 69 North Dongxia Road, Shantou, 515041, China; wenzhou@stu.edu.cn
3 Department of Materials Science & Engineering, Guangdong Provincial Key Laboratory of Energy Materials for Electric Power, Southern University of Science and Technology, Shenzhen 518055, China;
* Correspondence: yu.yang@scau.edu.cn (Y.Y.); zhanggz@sustech.edu.cn (G.Z.); yangzhuohong@scau.edu.cn (Z.Y.)
† These authors contributed equally to this work.

Supporting information

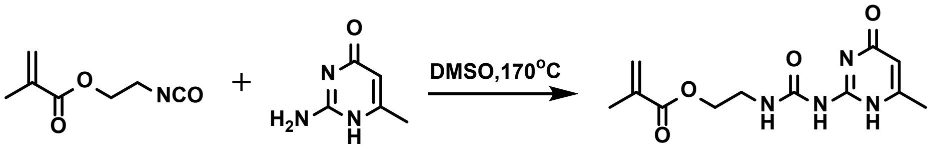

Scheme S1. Synthesis of UPy-MA monomer.

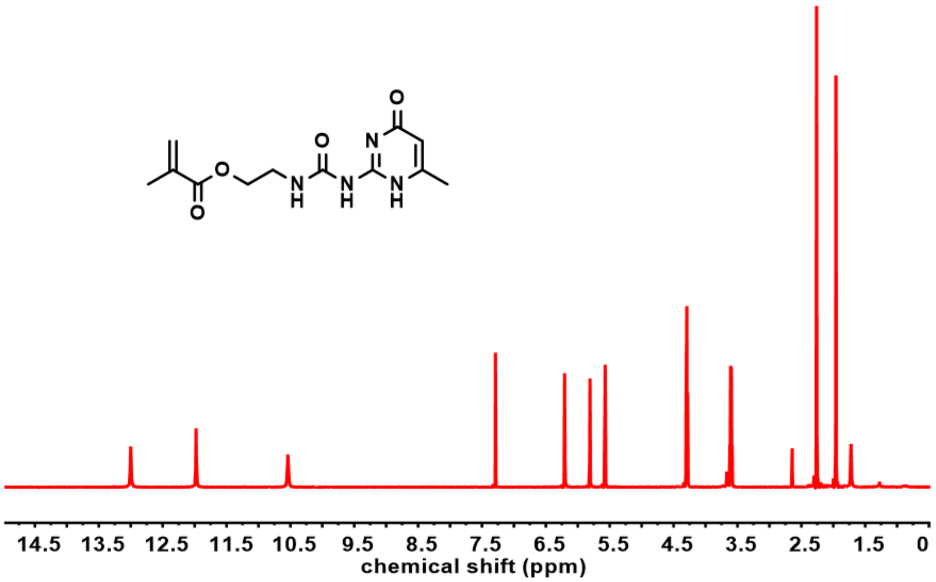

Figure S1. H NMR spectrum of UPy-NMA monomer.

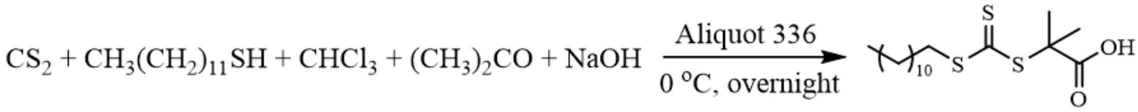

Figure S2. Synthesis of RAFT agent CTA.

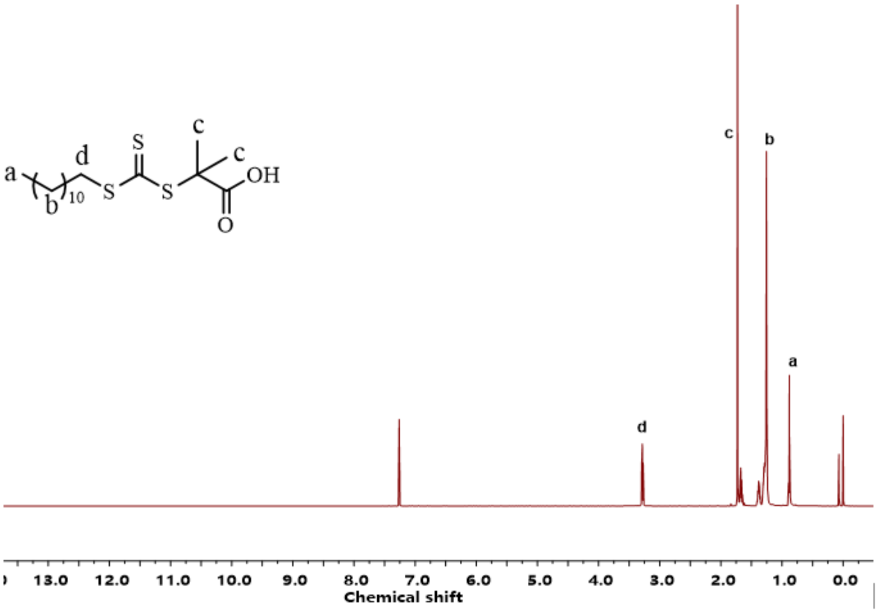

Figure S3. The H NMR spectrum of CTA.

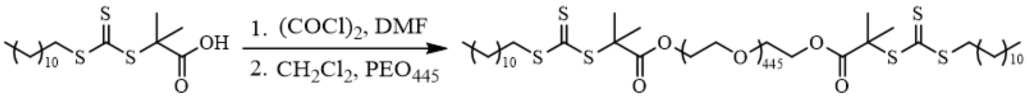

Figure S4. The synthesis of macro-RAFT agent (CTA-PEO-CTA).

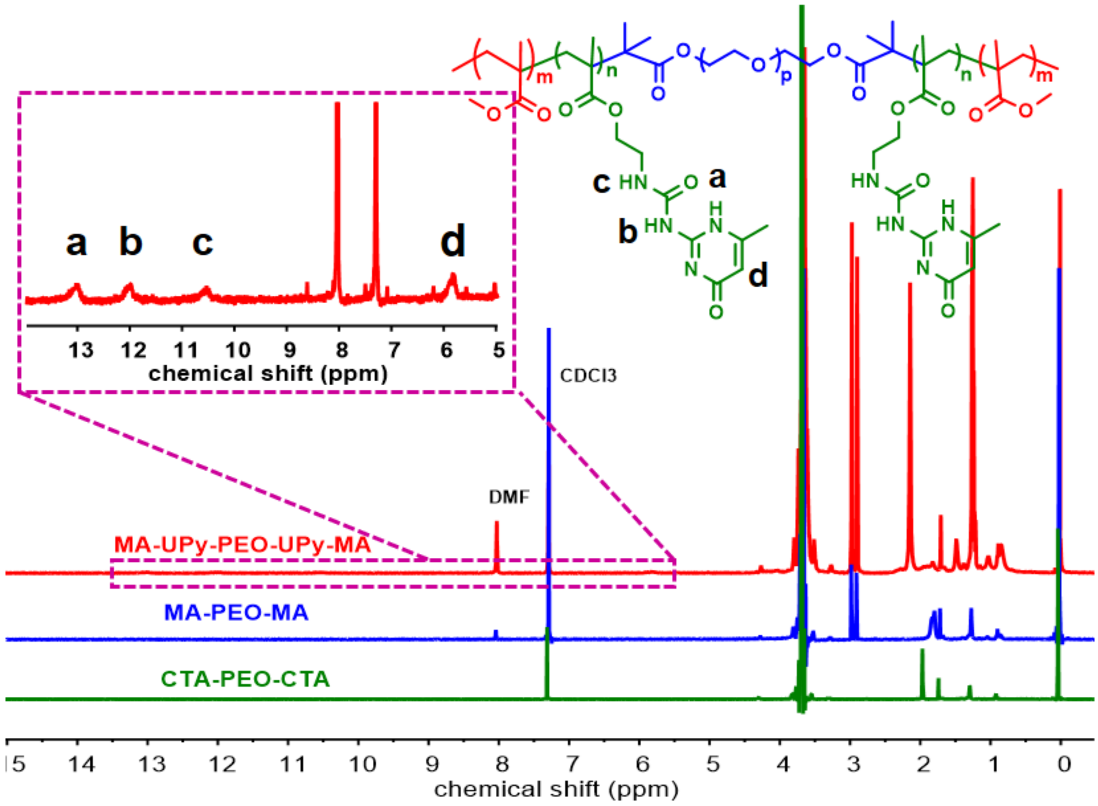

Figure S5. Characterizing the supramolecular polymers using H NMR.
